# The diagnostic accuracy of liver fibrosis in non-viral liver diseases using acoustic radiation force impulse elastography: A systematic review and meta-analysis

**DOI:** 10.1371/journal.pone.0227358

**Published:** 2020-01-15

**Authors:** Yuanqiang Lin, Hequn Li, Chunxiang Jin, Hui Wang, Bo Jiang

**Affiliations:** 1 Department of Ultrasound, China-Japan Union Hospital, Jilin University, China; 2 Department of General Surgery, Nanhu Hospital, China-Japan Union Hospital, Jilin University, China; Mayo Clinic College of Medicine, UNITED STATES

## Abstract

**Background:**

Acoustic radiation force impulse (ARFI) imaging is an ultrasound-based elastography method that has been studied in the staging of hepatic fibrosis, especially in chronic hepatitis. However, the diagnostic accuracy of ARFI in non-viral hepatopathies, such as autoimmune hepatitis and non-alcoholic fatty liver disease, has not been systematically determined.

**Aim:**

To systematically assess the diagnostic accuracy of ARFI in non-viral hepatopathies.

**Methods:**

The databases of PubMed, Embase, Cochrane Library and *clinicaltrials*.*gov* were systematically searched for candidate studies reporting the diagnostic accuracy of ARFI for hepatic fibrosis. The pooled estimates of the sensitivity, specificity, diagnostic odds ratio, and positive and negative likelihood ratios were calculated with the summary receiver operating curve (sROC) performed using STATA software.

**Results:**

In detail, a total of 29 diagnostic studies were included for further analysis. The quality of the included studies was relatively high using QUADAS method. The pooled sensitivity and specificity were 0.79 (0.73, 0.83) and 0.81 (0.75, 0.86), with AUROC 0.87 (0.83, 0.89) for the staging of significant fibrosis (F≥2). Meanwhile, for the staging of severe fibrosis (F≥3), the pooled sensitivity and specificity were 0.92 (0.87, 0.95) and 0.85 (0.80, 0.89), with AUROC 0.94 (0.92, 0.96). Furthermore, the pooled sensitivity and specificity were 0.89 (0.79, 0.95) and 0.89 (0.85, 0.92), with AUROC 0.94 (0.92, 0.96) for ARFI in staging cirrhosis (F = 4), which were similar to the data for severe fibrosis. No significant publication bias was present in this study.

**Conclusion:**

This meta-analysis demonstrated that ARFI exerted satisfactory diagnostic performance in staging non-viral hepatic fibrosis, especially severe fibrosis (F≥3) and cirrhosis (F = 4).

## Introduction

Hepatic fibrosis is one of the most common pathways for multiple insults, including viral infections, autoimmune factors, hereditary factors, metabolic and toxin-mediated hepatocellular dysfunctions [1]. The pathophysiological processes involve expansion of the extracellular matrix with liver fibrosis, then with portal hypertension and finally result in liver cirrhosis [2]. Previous studies have concluded that estimating the degree of liver fibrosis in patients with chronic liver disease (CLD) is important for disease surveillance, prognosis prediction, and appropriate treatment [3, 4].

Liver biopsy (LB) is considered the gold standard for liver fibrosis assessment. However, the primary limitation is the invasiveness of the procedure, which is not suitable in certain circumstances. Although the damage is quite “minimal”, the LB procedure still causes pain occurrence and minor or major bleeding (0.3%) and might lead to other complications even under ideal clinical conditions [5]. Meanwhile, the accuracy of liver biopsy in staging fibrosis is hampered by the intra-observer variability and sampling error [6, 7]. For instance, only 1/50000 of the whole liver tissue is sampled during a liver biopsy, for which sampling error is of concern [8]. The length of the specimens or the choice of the biopsy location might affect the accuracy of the results [9]. Moreover, repeated biopsies are infeasible in clinical settings for the sake of continuous monitoring of fibrosis grade [10]. Therefore, it is necessary to develop accurate and non-invasive methods to assess fibrosis stage and the progression of liver diseases to guide therapy.

Recent studies have indicated that several non-invasive techniques or parameters, including laboratory, radiological or ultrasonic techniques, have been developed for the accurate diagnosis of liver fibrosis. Laboratory parameters such as the aspartate aminotransferase to platelet ratio index (APRI)[11] and fibrosis-4 index (FIB-4) [12] have been studied in various disease models. Ultrasonography is the most commonly used diagnostic tool for preliminary screening of liver diseases. At present, several ultrasound systems have been developed to help improve the diagnostic performance in various diseases and include conventional color-Doppler ultrasound (CDUS), contrast-enhanced ultrasound (CEUS), acoustic radiation force impulse (ARFI) and transient elastography (TE) [13]. Specifically, ARFI elastography works first by B-mode imaging to locate a targeting region of interest (5 mm × 10 mm); then, the ARFI ultrasound probe is used to produce short pulses (approximately 262 microseconds with a frequency of 2.67 MHz) and generate shear waves tracked by ultrasound, which thus obtain a quantitative output in the form of shear wave velocity (SWV) [14, 15]. By using the short-duration acoustic radiation forces, which are less than 1 microseconds, the selected region of interest localizes the displacements without any external compression, and thus the operator dependency is reduced. The usage of ARFI could provide quantitative response during ultrasound tests [16]. Therefore, compared with conventional elastographic techniques, ARFI elastography provide quantitative measurements of SWV, which might be superior in evaluating the grade of hepatic fibrosis [14, 17]. The effects of ARFI elastography in staging hepatic fibrosis have been extensively studied with several meta analyses been published, especially regarding chronic hepatitis [1, 6, 17–19]. However, limited data are available on the performance of ARFI in staging liver fibrosis in non-alcoholic fatty/alcoholic liver diseases (NAFLD/ALD), non-alcoholic steatohepatitis (NASH), patients with liver transplantation or other autoimmune-related diseases (such as primary biliary cirrhosis, PBC), etc. Therefore, the aim of the current meta-analysis was to evaluate the overall diagnostic performance of ARFI imaging for the accurate grading of liver fibrosis in non-viral liver diseases by including all relevant publications and systematically calculating and analyzing the diagnostic data.

## Methods

### Literature search

Three databases of published articles (PubMed, Embase and the Cochrane Library) and unpublished data at www.clinicaltrials.gov were systematically searched for eligible articles in April 2019. No limits were placed on the publication date; each database was searched from inception to April 2019. During the searches, both medical subject headings (MESH) terms and other terms were used in different combinations. The search terms used included “hepatic”, “liver”, “fibrosis”, “cirrhosis”, “diagnosis”, “diagnostic”, “acoustic radiation force impulse” and “ARFI”, with the limitation of diagnostic studies. Two of the authors (Y Lin and H Li) performed the searching process independently, while a third investigator (B Jiang) solved any discrepancies during the study searching. The study protocols were approved by the Ethics Committee of China-Japan Union Hospital of Jilin University.

### Study selection

After searching the literature, inclusion and exclusion criteria were applied for further study identification. The studies were enrolled if they met the following criteria: (i) studies that concentrated on the diagnostic accuracy of ARFI for the staging of hepatic fibrosis in non-viral patients, i.e., studies using ARFI to evaluate the stages of hepatic fibrosis and assess the diagnostic accuracy of ARFI with the golden standard reference method of liver biopsy; (ii) studies that chose the well-recognized reference diagnostic standard and widely used methods for fibrosis staging, such as the LB plus METAVIR staging method [20]; (iii) studies that presented with direct values or indirect data to calculate the true-positive (TP), true-negative (TN), false-positive (FP) and false-negative (FN) values, for the construction of the 2×2 contingency table; and (iv) the reasons leading to hepatic fibrosis included cystic fibrosis-related liver disease, biliary atresia with or without operation, primary biliary cirrhosis, liver transplantation, non-alcoholic fatty liver disease, etc.

The studies were excluded if (i) they did not provide sufficient diagnostic data and one of the TP, FP, TN or FN values could not be deduced or calculated; (ii) the etiologies leading to hepatic fibrosis were virus infections, such as chronic hepatitis B, C or other subtypes; (iii) repeated or updated reports containing or overlapping with the same group of participants; and (iv) they were articles published in the forms of case reports, editorials, reviews or meta-analyses.

### Data extraction and quality assessment

Two of the investigators (Y Lin and H Li) were responsible for data extraction, and a third investigator (B Jiang) solved any discrepancies by group discussion. Information concerning the study publications, authors, year of publication, affiliated country, participant characteristics (number of patients, age and sex), reference method, methods for fibrosis staging, and TP, FP, TN and FN values were extracted. If no direct data of the diagnostic parameters were available, the values of these parameters were calculated backward through the values of the sensitivity, specificity, positive predictive value (PPV) or even the negative predictive value (NPV). The quality assessment of the included studies was conducted according to the Quality Assessment of Diagnostic Accuracy Studies (QUADAS) questionnaire [21].

### Data synthesis and statistical analysis

According to the METAVIR scoring system, hepatic fibrosis was staged into five groups (F0 = no fibrosis; F1 = portal fibrosis without septa; F2 = portal fibrosis and few septa, significant fibrosis; F3 = numerous septa without cirrhosis, severe fibrosis; and F4 = cirrhosis), and estimations of the diagnostic accuracy were made for the discrimination of F0 versus F1-4, F0/1 versus F2-4, F0-2 versus F3/4, and F0-3 versus F4. Here, these discriminations were presented in this meta-analysis as F≥1, F≥2, F≥3, and F≥4, respectively. In the data analysis, the bivariate random-effects model was used to estimate the pooled sensitivity, specificity, diagnostic score, diagnostic odds ratio (DOR), positive likelihood ratio (PLR) and the negative likelihood ratio (NLR), based on the sensitivity and specificity of each enrolled study. Meanwhile, a summary receiver operating characteristic (sROC) curve was calculated from all the included studies by adopting a weighted linear model. Furthermore, the area under the receiver operating curve (AUROC) in each drawn sROC curve was also used to evaluate the diagnostic accuracy of ARFI (a value of 0.9–1 was considered excellent, 0.8–0.9 was regarded as good, 0.7–0.8 as fair, 0.6–0.7 as poor and 0.5–0.6 as failed) [22]. The post-test probabilities were determined by the PLR and NLR values with the plot of the Fagan nomogram [23]. In addition, the Deeks’ method was adopted to assess the publication bias for analysis of more than ten studies [24]. The *I*^*2*^ statistic was used to assess the heterogeneity in the analysis of specificity and sensitivity. Values of 25, 50 and 75% for the *I*^*2*^ test were regarded as indicative of low, moderate and high statistical heterogeneity, respectively [25]. STATA version 14.0 software (StataCorp, College Station, TX) was used for the data analysis and graph-drawing, with *P*<0.05 regarded as significant. This meta-analysis was conducted according to the PRISMA checklist (S1 Table).

## Results

### Characteristics of included studies

**Fig 1:**
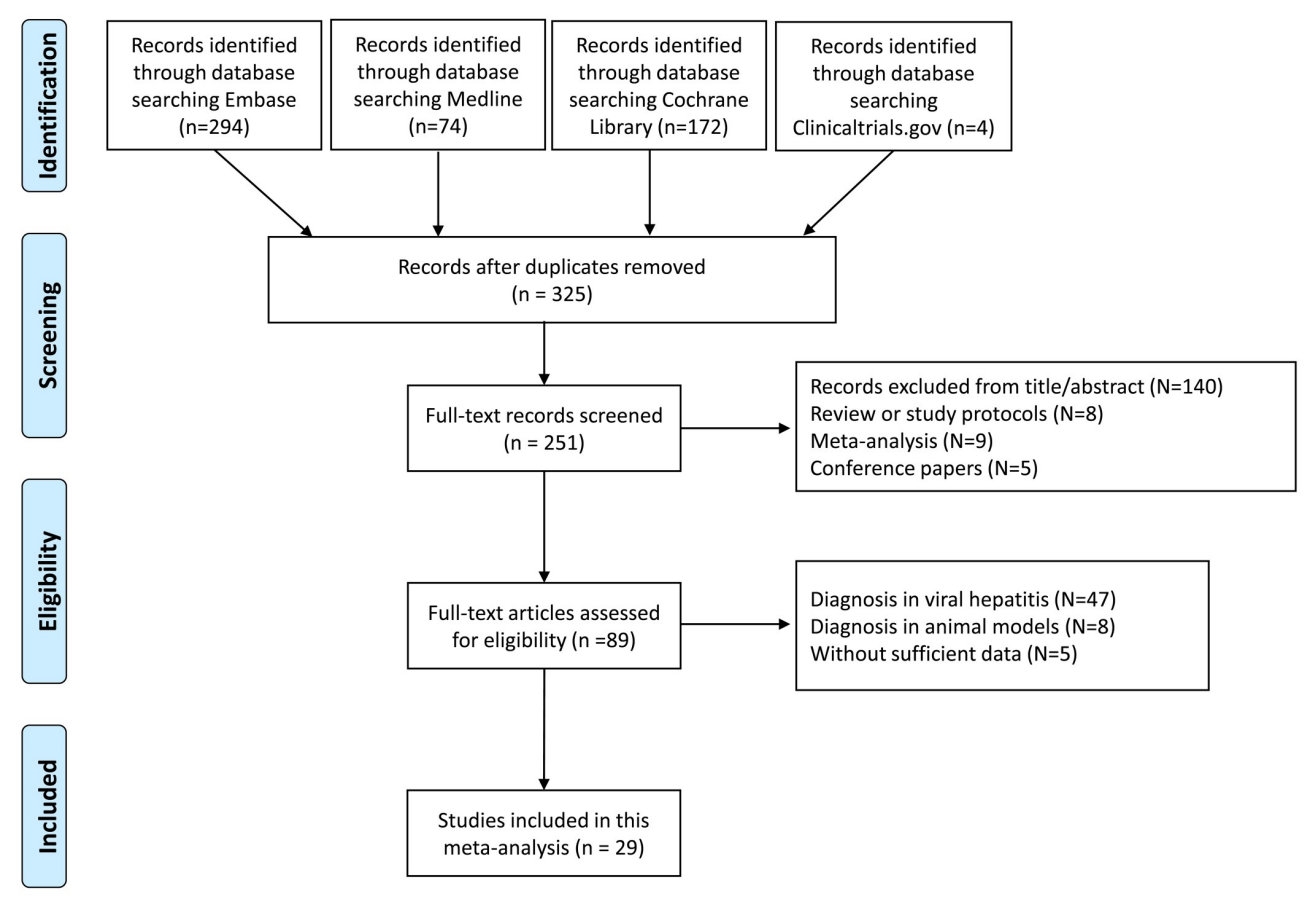
The flow diagram of literature searching and selection of studies according to the PRISMA criteria.

During the literature search, a total of 325 preliminary studies and 251 full text studies were identified for further screening (S1 Fig, S2 Table). After exclusion of the studies by title/abstract and other publication types, 89 studies were carefully assessed for eligibility. Then, the studies concentrating on viral hepatitis, animal models and those without sufficient data were excluded, and a total of 29 studies were included for further analysis [9, 26–53] (Fig 1). Among them, two studies concentrated on ARFI on biliary atresia-related hepatic fibrosis, two studies reported ARFI in diagnosing fibrosis caused by cystic fibrosis-associated liver disease. Additionally, seven studies were presented with liver transplantations, 15 studies reported liver fibrosis associated with NAFLD or NASH or ALD, and there was one comprehensive study with non-viral hepatic fibrosis. Table 1 presents the basic characteristics of the included studies, i.e., the number of patients, mean age and sex ratio, study region, gold standard, fibrosis staging methods and the diagnostic data, including the cut-off ARFI values, sensitivity, specificity and AUROC values of each stage. Furthermore, the mean age of patients included ranged from 59.7 months to 57 years. The fibrosis staging methods used included METAVIR, Batts-Ludwig’s system, Ludwig score, Kleiner’s method, Brunt’s staging system and Scheuer’s method.

**Table 1:** The basic characteristic of included studies.

Study and year	Disease model	Study region	Diagnostic criteria	Sample size	Mean age	Gender (M/F)	Treatment	Systems used	Stage	Cut off (m/s)	Sens	Spec	AUROC
Canas 2015	Cystic fibrosis	Spain	-	72	45	-	-	S2000	-	1.27	0.565	0.905	0.75 (0.61, 0.88)
Karlas 2012	Cystic fibrosis	Germany	-	55	31.9	31/24	-	S2000	-	1.28	0.429	0.925	-
Zhang 2014	PBC	China	Ludwig	56	45	10/46	-	S2000	≥F2 (39)	1.51	0.80	0.77	0.83 (0.72, 0.94)
									≥F3 (22)	1.79	0.91	0.82	0.93 (0.86, 0.99)
									= F4 (9)	2.01	1.00	0.79	0.91 (0.83, 0.99)
Zhang 2016	PBC	China	Child-Pugh	120	-	-	-	S2000	A (39)	-	0.579	0.933	-
									B (43)	-	0.974	0.75	-
									C (38)	-	0.921	0.833	-
Tomia 2016	biliary atresia	Japan	Metavir	22	6.3	-	-	S2000	≥F2 (17)	1.61	0.647	1	0.73 (0.50, 0.89)
									≥F3 (12)	1.70	0.833	1	0.91 (0.71, 0.99)
									= F4 (4)	2.00	1	0.833	0.86 (0.65, 0.97)
Gao 2017	biliary atresia	China	Batts-Ludwig	100	59.7 months	-	-	S2000	≥F2 (37)	1.53	0.914	0.615	0.82 (0.69, 0.92)
									≥F3 (19)	1.80	0.947	0.742	0.88 (0.76, 0.96)
									= F4 (8)	2.16	0.875	0.905	0.92 (0.80, 0.98)
Abdelhaleem 2018	Liver transplant	Egypt	Metavir	70	49.5	60/10	Immunosuppression regimens	S2000	≥F2 (28)	1.34	0.9	0.82	0.86
Liao 2014	Liver transplant	Taiwan	Metavir	57	57	43/14	-	S2000	≥F2 (3)	1.809	0.75	0.836	0.90 (0.78, 1)
									≥F3 (1)	2.331	1	0.929	0.93 (0.86, 1.00)
Pinto 2014	Liver transplant	Portugal	Batts and Ludwig	30	10.8	19/11	-	S2000	≥F2 (6)	-	0.83	0.58	0.4
Schmillevitch 2016	Liver transplant	Brazil	Metavir	33	55	22/11	-	S2000	≥F2 (12)	1.29	0.68	0.86	0.74 (0.55, 0.94)
									≥F3 (4)	1.42	0.75	0.86	0.77 (0.5, 1.0)
Tomita 2013	Liver transplant	Japan	Metavir	57	9.4	28/29	-	S2000	≥F2 (12)	1.30	0.75	0.818	0.85 (0.74, 0.96)
Wildner 2014	Liver transplant	Germany	Ludwig score	58	-	-	-	S2000	≥F2 (10)	1.75	0.64	0.82	0.73 (0.51, 0.95)
									≥F3 (5)	2.02	1	0.88	0.929 (0.819, 1)
Yoshino 2018	Liver transplant	Japan	Metavir	278	48.0	139/139	-	S2000	F2 (52)	1.31	0.894	0.533	-
									≥F3 (14)	1.53	0.929	0.697	-
Attia 2016	Obese, suspicious NAFLD	Germany	Kleiner	97	51.6	-	-	S2000	≥F2 (45)	1.17	0.86	0.87	0.90 (0.83, 0.97)
									≥F3 (27)	1.42	0.97	0.97	0.99 (0.96, 1.0)
									= F4 (17)	1.89	0.90	0.95	0.98 (0.96, 1.0)
Braticevici 2013	NAFLD	Romania	Metavir	84	48.5	36/48	-	S2000	≥F1 (43)	1.105	0.767	0.714	-
									≥F2 (33)	1.165	0.848	0.903	0.944
									≥F3 (22)	1.480	0.864	0.952	0.982
									= F4 (12)	1.635	0.917	0.923	0.984
Cassinotto 2016	NAFLD	France	Metavir	291	56.7	172/119	-	S2000	≥F2 (206)	1.32	0.56	0.91	0.77 (0.70, 0.83)
									≥F3 (126)	1.53	0.59	0.9	0.84 (0.78, 0.89)
									= F4 (49)	2.04	0.44	0.9	0.84 (0.78, 0.89)
Cui 2015	NAFLD	USA	NASH CRN	125	48.9	68/57	-	S3000	≥F1 (125)	1.29	0.542	0.774	0.66 (0.57, 0.76)
									≥F2 (33)	1.34	0.818	0.783	0.85 (0.78, 0.92)
									≥F3 (21)	1.34	0.952	0.740	0.90 (0.82, 0.97)
									= F4 (9)	2.48	0.778	0.931	0.86 (0.72, 1.0)
Friedrich-Rust 2012	NAFLD	Germany	Kleiner	57	45	30/27	-	S2000	≥F2 (16)	1.37	0.97	0.67	0.84
									≥F3 (11)	1.45	0.76	0.68	0.91
									= F4 (2)	1.75	0.74	0.67	0.91
Guerra 2015	NAFLD	Brazil	Metavir	24	51.4	11/13	-	-	≥F3 (11)	1.535	0.833	0.917	
Guzmán-Aroca 2012	NAFLD	Spain	Brunt	32	43.7	18/14	-	S2000	NASH or fibrosis	1.3	0.85	0.833	0.899
Harris 2016	NAFLD	Australia	Metavir	53	48.2	27/26	-	S2000	≥F2 (39)	1.48	0.74	0.58	0.65 (0.50, 0.80)
									≥F3 (30)	1.58	0.93	0.58	0.76 (0.61, 0.90)
Karlas 2015	Obese, suspicious NAFLD	Germany	NAS staging	41	45.7	28/13	14-day low-energy diet	S2000	≥F2 (8)	-	0.83	0.79	
Lee 2017	NAFLD	Korea	Brunt	94	55.5	41/53	-	S2000	≥F2 (46)	-	0.462	0.932	0.66 (0.55, 0.76)
									≥F3 (27)	-	0.7	0.937	0.87 (0.78, 0.97)
									= F4 (14)	-	0.75	0.907	0.92 (0.85, 0.99)
Osaki 2010	NASH	Japan	Brunt	23	-	-	-	S2000	≥F3 (13)	2.20	1	0.75	0.942
Palmeri 2011	NAFLD	USA	Metavir	172	-	65/107	-	S2000	≥F3	2.06	0.9	0.9	0.9
Yoneda 2010	NAFLD	Japan	Brunt	54	50.6	25/29	-	S2000	≥F3 (13)	1.77	1	0.91	0.93
									= F4 (5)	1.9	1	0.96	0.97
Kiani 2016	ALD	France	Metavir	82	43.8	69/13	-	S2000	≥F2 (34)	1.63	0.824	0.833	0.87
									≥F3 (17)	1.84	0.824	0.785	0.86
									= F4 (13)	1.94	0.923	0.816	0.89
Zhang 2015	ALD	China	Scheuer	99	40.7	93/6	-	S2000	≥S2 (60)	1.27	0.77	0.85	0.85 (0.77, 0.92)
									≥S3 (25)	1.40	0.84	0.82	0.88 (0.79, 0.96)
									= S4 (9)	1.65	0.89	0.84	0.89 (0.82, 0.96)
Park 2017	Non-viral liver diseases	-	Metavir	199	-	-	-	-	≥F2	-	0.802	0.591	0.85 (0.76, 0.94)
									= F4	-	0.565	0.965	-

**Abbreviations**: PBC, primary biliary cirrhosis; NAFLD, non-alcoholic fatty liver disease; ALD, alcoholic liver disease.

### Quality assessment

Before the data analysis and synthesis, the quality of eligible studies was evaluated using the QUADAS questionnaire, presented in Table 2. Regarding the judging criteria, high study quality was defined when at least 9 of the 14 items in the checklist were considered “yes”. In detail, as to item 2, concerning whether the inclusion criteria were clearly described, a total of 11 studies answered “no” with the other 18 studies answering “yes”. As for item 8, concerning whether the protocol of the index test described was sufficient to completely replicate the test, it was rated “no” for 4 studies. Regarding item 9, with respect to whether the particulars of the reference standard were clearly elucidated, it was shown that 7 studies answered “no”. With regard to the remaining items, all the studies included answered “yes”. Therefore, most of the included studies were rated as high quality.

**Table 2:** Quality assessment of the studies included in our meta-analysis.

Publication and reference number	Item number on QUADAS Tool													
	Representative spectrum	Description of study criteria	Acceptable reference standard	Disease progression bias avoided	Partial verification bias avoided	Differential verification bias avoided	Incorporation bias avoided	Description of index test	Description of reference standard	Blinding to reference	Blinding to index test	Availability of clinical data	Uninterpretable results reported	Withdrawals explained
Canas 2015	Yes	No	Yes	Yes	Yes	Yes	Yes	Yes	No	Yes	Yes	Yes	Yes	Yes
Karlas 2012	Yes	Yes	Yes	Yes	Yes	Yes	Yes	Yes	No	Yes	Yes	Yes	Yes	Yes
Zhang 2014	Yes	Yes	Yes	Yes	Yes	Yes	Yes	Yes	Yes	Yes	Yes	Yes	Yes	Yes
Zhang 2016	Yes	Yes	Yes	Yes	Yes	Yes	Yes	Yes	No	Yes	Yes	Yes	Yes	Yes
Tomia 2016	Yes	No	Yes	Yes	Yes	Yes	Yes	Yes	Yes	Yes	Yes	Yes	Yes	Yes
Gao 2017	Yes	Yes	Yes	Yes	Yes	Yes	Yes	Yes	Yes	Yes	Yes	Yes	Yes	Yes
Abdelhaleem 2018	Yes	Yes	Yes	Yes	Yes	Yes	Yes	Yes	Yes	Yes	Yes	Yes	Yes	Yes
Liao 2014	Yes	No	Yes	Yes	Yes	Yes	Yes	Yes	Yes	Yes	Yes	Yes	Yes	Yes
Pinto 2014	Yes	No	Yes	Yes	Yes	Yes	Yes	Yes	Yes	Yes	Yes	Yes	Yes	Yes
Schmillevitch 2016	Yes	No	Yes	Yes	Yes	Yes	Yes	No	Yes	Yes	Yes	Yes	Yes	Yes
Tomita 2013	Yes	No	Yes	Yes	Yes	Yes	Yes	Yes	Yes	Yes	Yes	Yes	Yes	Yes
Wildner 2014	Yes	No	Yes	Yes	Yes	Yes	Yes	Yes	Yes	Yes	Yes	Yes	Yes	Yes
Yoshino 2018	Yes	No	Yes	Yes	Yes	Yes	Yes	Yes	Yes	Yes	Yes	Yes	Yes	Yes
Attia 2016	Yes	No	Yes	Yes	Yes	Yes	Yes	Yes	Yes	Yes	Yes	Yes	Yes	Yes
Braticevici 2013	Yes	Yes	Yes	Yes	Yes	Yes	Yes	Yes	Yes	Yes	Yes	Yes	Yes	Yes
Cassinotto 2016	Yes	Yes	Yes	Yes	Yes	Yes	Yes	Yes	No	Yes	Yes	Yes	Yes	Yes
Cui 2015	Yes	Yes	Yes	Yes	Yes	Yes	Yes	Yes	Yes	Yes	Yes	Yes	Yes	Yes
Friedrich-Rust 2012	Yes	No	Yes	Yes	Yes	Yes	Yes	Yes	Yes	Yes	Yes	Yes	Yes	Yes
Guerra 2015	Yes	No	Yes	Yes	Yes	Yes	Yes	No	No	Yes	Yes	Yes	Yes	Yes
Guzmán-Aroca 2012	Yes	Yes	Yes	Yes	Yes	Yes	Yes	Yes	Yes	Yes	Yes	Yes	Yes	Yes
Harris 2016	Yes	No	Yes	Yes	Yes	Yes	Yes	Yes	Yes	Yes	Yes	Yes	Yes	Yes
Karlas 2015	No	Yes	Yes	Yes	Yes	Yes	Yes	Yes	Yes	Yes	Yes	Yes	Yes	Yes
Lee 2017	Yes	Yes	Yes	Yes	Yes	Yes	Yes	Yes	Yes	Yes	Yes	Yes	Yes	Yes
Osaki 2010	Yes	Yes	Yes	Yes	Yes	Yes	Yes	Yes	No	Yes	Yes	Yes	Yes	Yes
Palmeri 2011	Yes	Yes	Yes	Yes	Yes	Yes	Yes	No	Yes	Yes	Yes	Yes	Yes	Yes
Yoneda 2010	Yes	Yes	Yes	Yes	Yes	Yes	Yes	Yes	Yes	Yes	Yes	Yes	Yes	Yes
Kiani 2016	Yes	Yes	Yes	Yes	Yes	Yes	Yes	Yes	Yes	Yes	Yes	Yes	Yes	Yes
Zhang 2015	Yes	Yes	Yes	Yes	Yes	Yes	Yes	Yes	Yes	Yes	Yes	Yes	Yes	Yes
Park 2017	Yes	Yes	Yes	Yes	Yes	Yes	Yes	No	No	Yes	Yes	Yes	Yes	Yes
No. of “Yes” answers	29	18	29	29	29	29	29	25	22	29	29	29	29	29
No. of “No” answers	0	11	0	0	0	0	0	4	7	0	0	0	0	0

### Diagnostic accuracy of ARFI imaging in significant fibrosis (F≥2)

**Fig 2:**
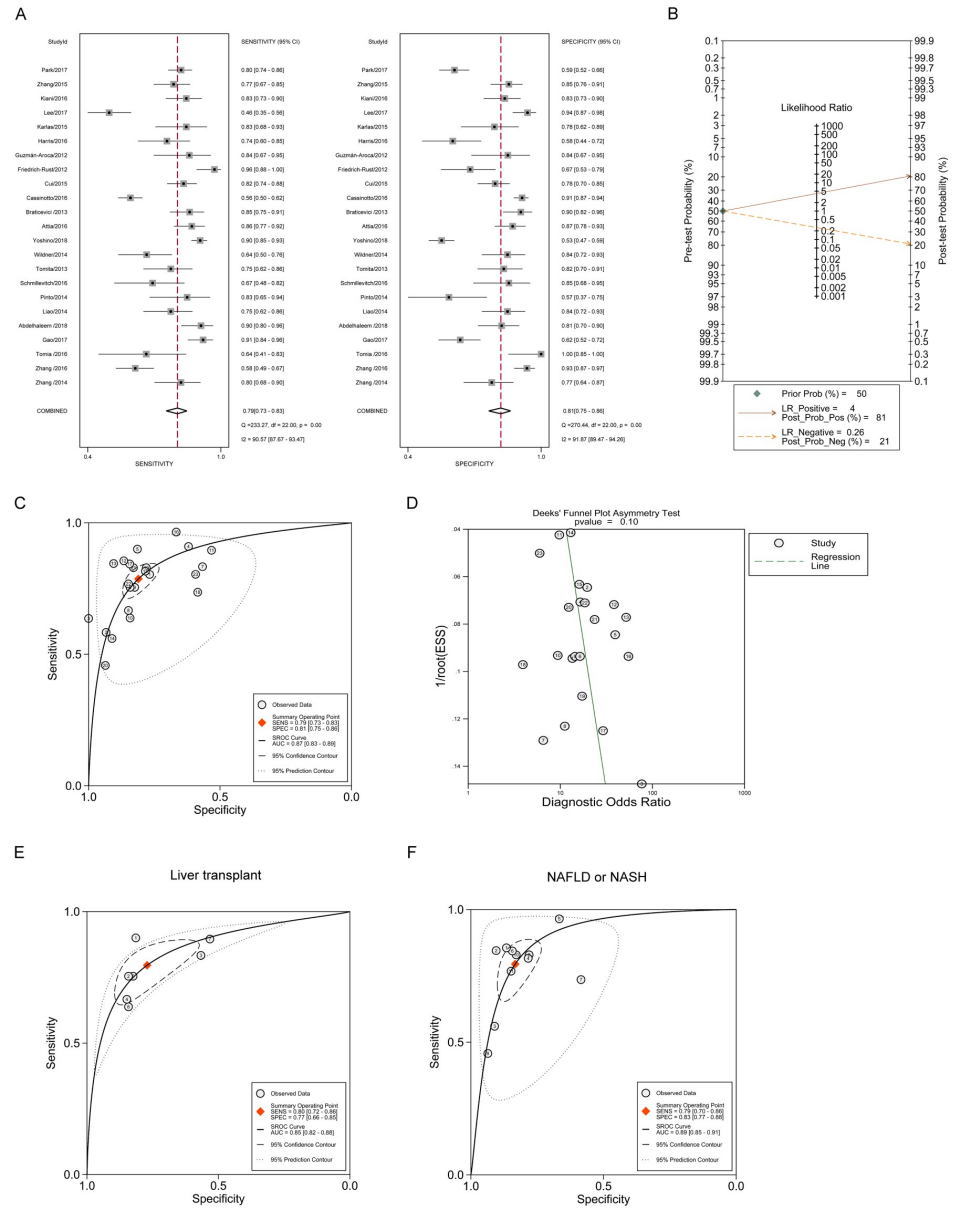
The diagnostic performance of ARFI in staging the significant fibrosis (F≥2). (A) Forest plot for the pooled estimates of sensitivity and specificity of ARFI on the differentiation of significant fibrosis. (B) Fagan nomogram for the differentiation of significant hepatic fibrosis with ARFI. (C) The summary receiver operating curve (SROC) and corresponding area under ROC (AUROC) for the differentiation of significant hepatic fibrosis with ARFI. (D) Deeks’ funnel plot for the assessment of publication bias. (E) The sROC curve and corresponding AUROC for the differentiation of significant hepatic fibrosis with ARFI in patients with liver transplant. (F) SROC curve and corresponding AUROC for the differentiation of significant hepatic fibrosis with ARFI in patients with non-alcoholic fatty / alcoholic liver diseases (NAFLD or NASH).

Using the bivariate model, it is shown in Fig 2A that the pooled sensitivity estimate was 0.79 with 95% confidence interval (CI) (0.73, 0.83), and the pooled specificity estimate was 0.81 with 95% CI (0.75, 0.86). The *I*^*2*^ statistics for sensitivity analysis and specificity analysis were 90.57 (87.67, 93.47) and 91.87 (89.47, 94.26). Additionally, the pooled diagnostic score with 95% CI was 2.76 (2.49, 3.03) with DOR 15.77 (12.04, 20.65). The pooled PLR and NLR, with their 95% CIs, were 4.15 (3.28, 5.26) and 0.26 (0.22, 0.32), respectively. Meanwhile, the Fagan nomogram showed that the ARFI values larger than the cut-off value increased the pretest probability of significant fibrosis from 50% to 81% of the posttest probability for a positive test result, whereas a smaller ARFI value decreased the pretest probability from 50% to 21% of the posttest probability for a negative test result (Fig 2B). The whole sROC curve was plotted and the AUROC with 95% CI was 0.87 (0.83, 0.89) (Fig 2C). Furthermore, the publication bias was assessed in Fig 2D using Deeks’ funnel plot asymmetry test, demonstrating that the study numbers on both sides of the regression line were similar and no significant publication bias was observed with P value 0.10. These results showed that ARFI imaging exhibited good performance in differentiating significant fibrosis. Additionally, the subgroup analysis was conducted to further identify the role of ARFI imaging in certain diseases. Fig 2E shows that the AUROC using ARFI was 0.85 (0.82, 0.88) in patients with liver transplantation, whereas Fig 2F demonstrates that the AUROC with 95% CI was 0.89 (0.85, 0.91) in NAFLD or NASH patients. Meanwhile, the cutoff values were analyzed. The cutoff values ranged from 1.53–1.61 m/s for biliary atresia, 1.29–1.809 m/s for liver transplant, and 1.105–1.48 m/s for NAFLD, indicating that the cutoff values had high consistency with F2 staging. More specifically, the cutoff values from sROC curve were analyzed in Table 3. It was shown that the cutoff value was 1.34 m/s for significant fibrosis (F≥2) staging as a whole, 1.30 m/s for both liver transplant and NAFLD subgroup analyses. The differences of cutoff values between these disease models might contribute to the heterogeneity between studies.

**Table 3:** The summary cutoff values of the enrolled studies.

Diagnosis	Cutoff values (m/s)	Sensitivity	Specificity
F≥2	1.34	0.81	0.78
F≥2 (Liver transplant)	1.30	0.75	0.81
F≥2 (NAFLD)	1.30	0.85	0.83
F≥3	1.79	0.91	0.82
F≥3 (Liver transplant)	2.02	1	0.88
F≥3 (NAFLD)	2.06	0.9	0.9
F = 4	2.16	0.88	0.91
F = 4 (NAFLD)	1.89	0.90	0.95

### Diagnostic accuracy of ARFI imaging in severe fibrosis (F≥3)

**Fig 3:**
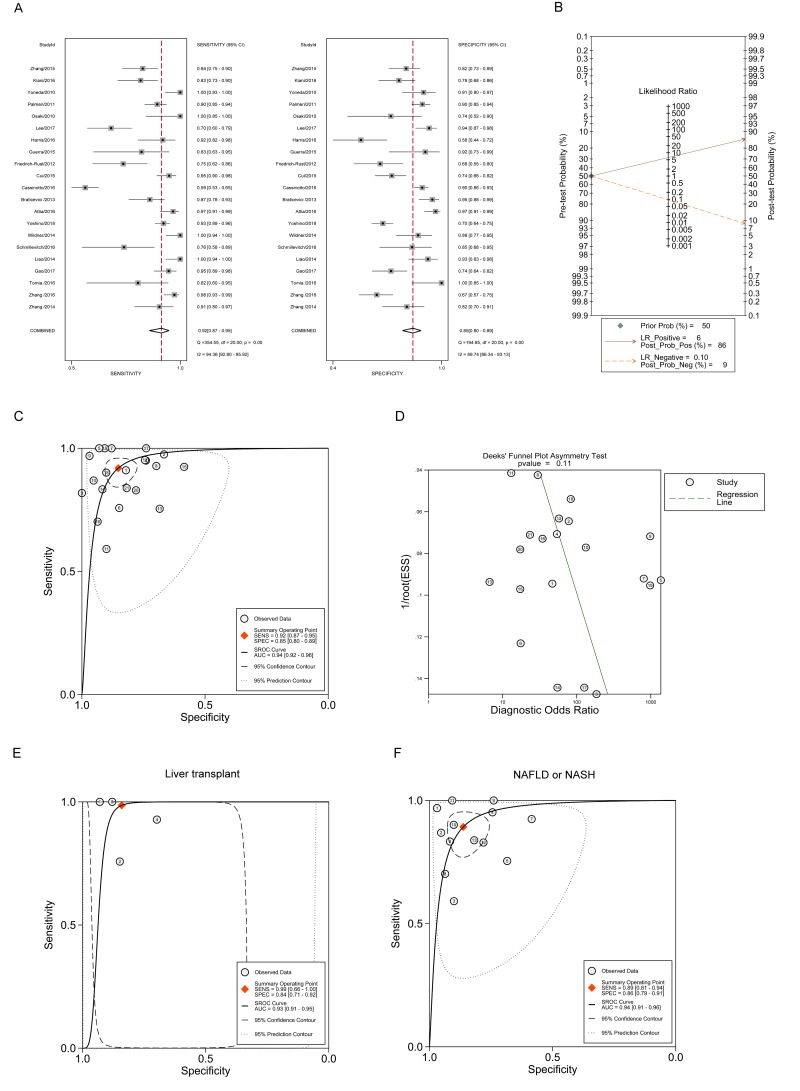
The diagnostic performance of ARFI in staging severe fibrosis (F≥3). (A) Forest plot for the pooled estimates of sensitivity and specificity of ARFI on the differentiation of severe fibrosis. (B) Fagan nomogram for the differentiation of severe hepatic fibrosis with ARFI. (C) The summary receiver operating curve (SROC) and corresponding area under ROC (AUROC) for the differentiation of severe hepatic fibrosis with ARFI. (D) Deeks’ funnel plot for the assessment of publication bias. (E) SROC curve and corresponding AUROC for the differentiation of severe hepatic fibrosis with ARFI in patients with liver transplant. (F) SROC curve and corresponding AUROC for the differentiation of severe hepatic fibrosis with ARFI in patients with NAFLD or NASH.

Regarding ARFI imaging in staging severe fibrosis adopting the bivariate model, Fig 3A shows that the pooled sensitivity estimate was 0.92 with 95% CI (0.87, 0.95), and the specificity estimate was 0.85 with 95% CI (0.80, 0.89). The *I*^*2*^ statistics for sensitivity analysis and specificity analysis were 94.36 (92.80, 95.92) and 89.74 (86.34, 93.13). Additionally, the pooled diagnostic score with 95% CI was 4.18 (3.54, 4.83) with DOR 65.57 (34.36, 125.13). The pooled PLR and NLR, with their 95% CIs, were 6.23 (4.51, 8.59) and 0.10 (0.06, 0.16), respectively. Meanwhile, the Fagan nomogram showed that the ARFI values larger than the cut-off value increased the pretest probability of severe fibrosis from 50% to the posttest probability of 86% for a positive test result, whereas a smaller ARFI value decreased the pretest probability from 50% to the posttest probability of 9% for a negative test result (Fig 3B). The whole sROC curve was plotted and the AUROC with 95% CI was 0.94 (0.92, 0.96) (Fig 3C). Furthermore, the publication bias was assessed in Fig 3D, demonstrating that a relatively similar number of studies were distributed between the regression line and that no significant publication bias was observed with a P value of 0.11. These results showed that ARFI imaging exhibited excellent performance in differentiating severe fibrosis, better than significant fibrosis. Additionally, a subgroup analysis was conducted, with Fig 3E showing that the AUROC using ARFI was 0.93 (0.91, 0.95) in patients with liver transplantation, whereas Fig 3F demonstrates that the AUROC with 95% CI was 0.94 (0.91, 0.96) in NAFLD or NASH patients. For the cutoff values, the cutoff values ranged from 1.70–1.80 m/s for biliary atresia, 1.42–2.331 m/s for liver transplant, and 1.34–2.20 m/s for NAFLD. For liver transplant and NAFLD, the cutoff values had a relatively large range, which might be the main factor contributed to the high heterogeneity between studies. As to the cutoff values in the sROC curve, it was shown that the cutoff value is 1.79 m/s for severe fibrosis (F≥3) staging as a whole, and 2.02 and 2.06 m/s for the subgroups of liver transplant and NAFLD, respectively.

### Diagnostic accuracy of ARFI imaging in cirrhosis (F = 4)

**Fig 4:**
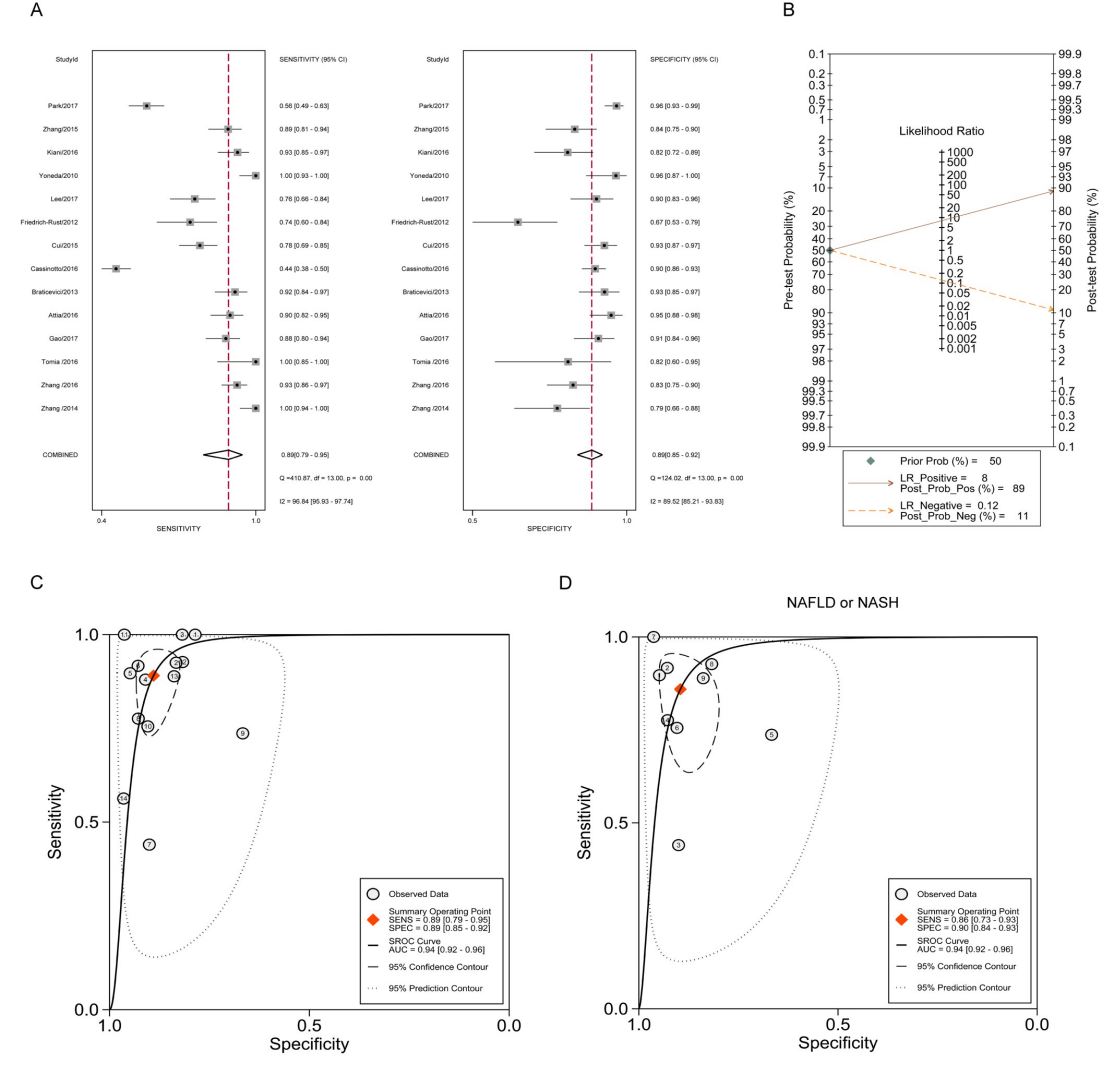
The diagnostic performance of ARFI in staging cirrhosis (F = 4). (A) Forest plot for the pooled estimates of sensitivity and specificity of ARFI on the differentiation of cirrhosis. (B) Fagan nomogram for the differentiation of hepatic cirrhosis with ARFI. (C) The summary receiver operating curve (SROC) and corresponding area under ROC (AUROC) for the differentiation of hepatic cirrhosis with ARFI. (D) SROC curve and corresponding AUROC for the differentiation of hepatic cirrhosis with ARFI in patients with NAFLD or NASH.

As for the ARFI imaging in staging hepatic cirrhosis, Fig 4A shows that the pooled sensitivity estimate was 0.89 with 95% CI (0.79, 0.95), and the specificity estimate was 0.89 with 95% CI (0.85, 0.92). The *I*^*2*^ statistics for sensitivity analysis and specificity analysis were 96.84 (95.93, 97.74) and 89.52 (85.21, 93.83). Additionally, the pooled diagnostic score with 95% CI was 4.19 (3.39, 4.98) with DOR 65.81 (29.62, 146.20). The pooled PLR and NLR, with their 95% CIs were 8.09 (5.79, 11.31) and 0.12 (0.06, 0.24), respectively. Meanwhile, the Fagan nomogram showed that the ARFI values larger than the cut-off value increased the pretest probability of cirrhosis from 50% to the posttest probability of 89% for a negative test result, whereas a smaller ARFI value decreased the pretest probability from 50% to the posttest probability of 11% for a negative test result (Fig 4B). The whole sROC curve was plotted and the AUROC with 95% CI was 0.94 (0.92, 0.96), which was the same as that for the F3 staging (Fig 4C). Additionally, a subgroup analysis was conducted, with Fig 4D showing that the AUROC using ARFI was 0.94 (0.92, 0.95) in patients with NAFLD or NASH. The cutoff values ranged from 2.0–2.16 m/s for biliary atresia. For NAFLD, the cutoff values ranged from 1.635–2.48 m/s because different disease progressions were observed in these studies. For the cutoff values from the sROC curve, it was shown that 2.16 m/s as a whole for cirrhosis (F = 4) staging and 1.89 m/s for NAFLD subgroup. In summary, these results showed that ARFI imaging exhibited better performance in differentiating severe fibrosis and cirrhosis, which was better than that in staging significant fibrosis.

## Discussion

In this meta-analysis, we analyzed the diagnostic accuracy of ARFI in hepatic fibrosis staging in non-viral hepatopathy by searching and including all the relevant and eligible diagnostic studies. It was demonstrated that for the staging of significant fibrosis (F≥2), the pooled sensitivity and specificity were 0.79 (0.73, 0.83) and 0.81 (0.75, 0.86), with AUROC 0.87 (0.83, 0.89). Meanwhile, for the staging of severe fibrosis (F≥3), the pooled sensitivity and specificity were 0.92 (0.87, 0.95) and 0.85 (0.80, 0.89), with AUROC 0.94 (0.92, 0.96). Additionally, the diagnostic score and DOR values showed that ARFI exhibited better performance in differentiating severe fibrosis. Furthermore, the pooled sensitivity and specificity were 0.89 (0.79, 0.95) and 0.89 (0.85, 0.92), with AUROC 0.94 (0.92, 0.96) for ARFI in staging cirrhosis (F = 4), which were similar to the data in severe fibrosis. In addition, the subgroup analyses concerning the diagnostic performances of ARFI in patients with liver transplant or NAFLD were also presented, indicating similar results as the total analysis. No significant publication bias was present. Therefore, our study demonstrated that ARFI exerted better diagnostic performance in staging hepatic fibrosis, especially in severe fibrosis (F≥3) and cirrhosis (F = 4).

The diagnostic performance of ARFI in hepatic fibrosis caused by various diseases has been studied widely. A previous study indicated that a diagnostic tool is regarded as perfect if the AUROC rate is 100%, excellent if the AUROC value is greater than 90% and good if the AUROC is larger than 80% [54, 55]. Generally, when diagnosing a higher stage of liver fibrosis, i.e., over stage 3 (F≥3), ARFI elastography has a higher sensitivity, specificity, and AUROC values than those of the F≥2 and F≥1 liver fibrosis stage, which was determined to be the basic features of ARFI. For chronic viral hepatitis, Hu et al. [18] reported that ARFI elastography is an accurate and reliable method for the diagnosis of both chronic hepatitis B- and chronic hepatitis C-mediated liver fibrosis, especially for the evaluation of stages F≥3 and F = 4. These results are highly consistent with our study, although limited data are available in non-viral hepatopathies. In our studies, we also enrolled studies reporting the diagnostic role of ARFI in PBC and cystic fibrosis-related liver fibrosis (CFLD), which have been reported in several studies. Due to data insufficiency, only four available studies were included in the final data analysis reporting these two diseases, and no subgroup analysis was conducted. For example, Cañas et al. [28] reported that ARFI elastography was useful for detecting CFLD and has the potential to be a useful tool to noninvasively evaluate liver involvement and disease progression. Meanwhile, for the diagnosis in PBC, Zhang reported that the AUROC values of ARFI elastography for predicting histological stages equal to or higher than stage II, stage III and equal to stage IV were 0.83, 0.93, and 0.91, respectively [49]. Due to the limited number of studies and data availability, no further analysis concerning PBC was conducted. However, a recent systematic review summarized the non-invasive diagnosis of autoimmune-related hepatic fibrosis, demonstrating that the transient elastography technique has good performance in staging liver fibrosis in patients with autoimmune hepatitis, whereas the diagnostic accuracy of APRI is inferior [10]. Therefore, the diagnostic accuracy of ARFI in autoimmune hepatopathies still needs further investigation. Furthermore, we analyzed the diagnostic efficacy of ARFI in NAFLD or NASH, showing that the AUROCs were 0.89 (0.85, 0.91) for staging significant fibrosis (F≥2) and 0.94 (0.91, 0.96) for staging severe fibrosis (F≥3) in NAFLD or NASH patients, which is similar to a previous meta-analysis [17], but we enrolled more studies and had more comprehensive analysis.

As to the cutoff values for staging hepatic fibrosis, several studies have provided clues and values to help improve the clinical decision. As early as the year 2011, Friedrich-Rust et al. reported the different cutoff values for liver fibrosis staging [32]. The values are 1.34 m/s for significant fibrosis (F≥2), 1.55 m/s for severe fibrosis (F≥3) and 1.80 m/s for cirrhosis (F = 4) from 518 patients. It was shown that the cutoff values for F≥3 and F = 4 are higher in our analysis compare with those of Friedrich-Rust et al., which also improved the specificity of the diagnosis. However, the studies of Nierhoff et al. [6], Bota et al. [19], Guo et al. [22], Liu et al. [17] and Wu et al. [10] did not report the cutoff values from the sROC curve. In addition, Hu et al. concentrated on ARFI on fibrosis staging in chronic hepatitis B and C patients, showing that that the chronic hepatitis C patients had higher combined ARFI values compared with hepatitis B patients [18]. Therefore, the results of our study provided a pooled analysis from 27 studies and gave the appropriate cutoff values for fibrosis staging in non-viral liver diseases.

On the other hand, a majority of studies including mixed disease models have been reported. For example, Dhyani et al. reported that ARFI had good diagnostic performance (AUROC 0.92) for the diagnosis of significant fibrosis at a depth of 7 cm along the central axis in diffuse liver diseases [56]. Gani et al. [57] reported the diagnostic accuracy of ARFI on significant fibrosis (F≥2) with the an AUROC value of 0.773 (0.616, 0.930) and an even better accuracy for diagnosing cirrhosis (F4 fibrosis, AUROC 0.856 (0.736, 0.975)), which is inferior to our study. The difference may be attributed to the study population selection and the different disease models. A recent study by Pfeifer et al. [58] demonstrated that combining ARFI elastography with other noninvasive tests in patients with suspected liver disease could significantly increase the diagnostic accuracy for liver cirrhosis compared with ARFI alone. Therefore, which combination has the best diagnostic accuracy is a new study direction in the future.

This diagnostic meta-analysis has several limitations. First, ARFI elastography is a relatively newly developed technology that has not been widely investigated in autoimmune hepatopathy or other disease models. Due to the limitation of the study number and data availability, a subgroup analysis in several disease models was not conducted. Second, heterogeneity analysis was not conducted, although no significant publication bias was observed. Third, the details of liver biopsy are not fully reported in several studies, and the staging systems of hepatic fibrosis are not consistent, which might increase the heterogeneity and decrease the reliability of the diagnostic performance of ARFI in hepatic fibrosis.

## Conclusion

In summary, our meta-analysis indicates that ARFI imaging has good performance in staging hepatic fibrosis in non-viral patients, especially in severe fibrosis and cirrhosis.

## Supporting information

S1 Fig Flow diagram of the searching process. (TIF)

S1 Table PRISMA checklist. (DOC)

S2 Table The searching strategy of the MEDLINE database. (DOCX)
