# Supplementary figures and images for: The diagnostic accuracy of liver fibrosis in non-viral liver diseases using acoustic radiation force impulse elastography: A systematic review and meta-analysis
Source: PLoS One. 2020 Jan 15;15(1):e0227358. doi: 10.1371/journal.pone.0227358 (PMC6961899; doi:10.1371/journal.pone.0227358)

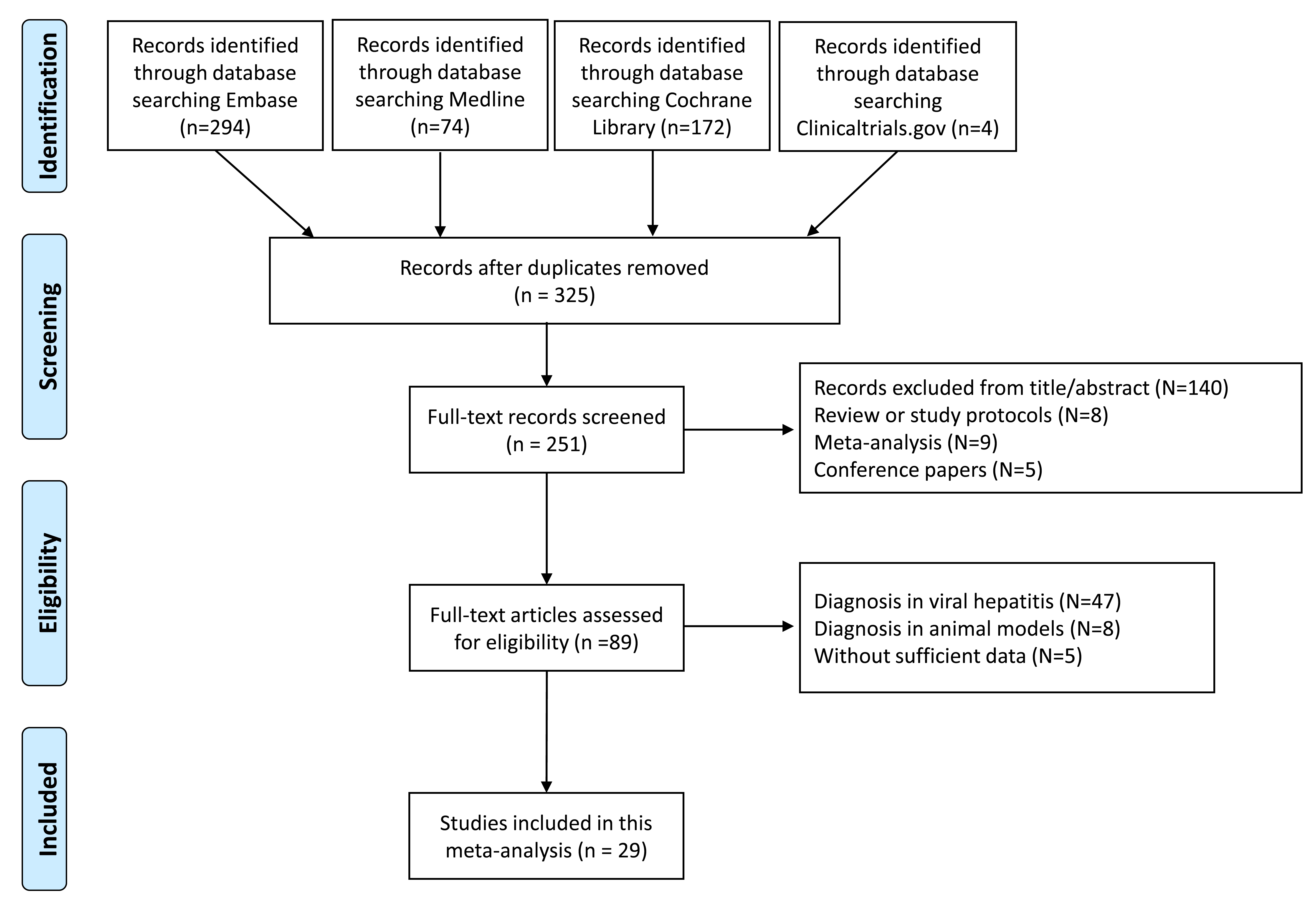

Supplement: S1 Fig — (TIF) [file pone.0227358.s001.tif]
