# Supplementary material for: The diagnostic accuracy of liver fibrosis in non-viral liver diseases using acoustic radiation force impulse elastography: A systematic review and meta-analysis
Source: PLoS One. 2020 Jan 15;15(1):e0227358. doi: 10.1371/journal.pone.0227358 (PMC6961899; doi:10.1371/journal.pone.0227358)
Supplement: S2 Table — (DOCX) [file pone.0227358.s003.docx]

| #1 “hepatic” [Title/Abstract] |
| --- |
| #2 “liver” [MESH] |
| #3 #1 OR #2 |
| #4 “fibrosis” [MESH] |
| #5 “Liver cirrhosis” [MESH] |
| #6 #4 OR #5 |
| #7 “Elasticity Imaging Techniques” [MESH] |
| #8 “Technique, Elasticity Imaging” [MESH] |
| #9 “Acoustic radiation force impulse” [Title/Abstract] |
| #10 “ARFI” [Title/Abstract] |
| #11 “ARFI imaging” [MESH] |
| #12 #7 OR #8 OR #9 OR #10 OR #11 |
| #13 “diagnosis” [MESH] |
| #14 “diagnostic” [Title/Abstract] |
| #15 “diagnostic study” [Title/Abstract] |
| #16 #13 OR #14 OR #15 |
| #17 #3 AND #6 AND #12 AND #16 |
